# Microbiological Evaluation and Sperm DNA Fragmentation in Semen Samples of Patients Undergoing Fertility Investigation

**DOI:** 10.3390/genes12050654

**Published:** 2021-04-27

**Authors:** Chiara Pagliuca, Federica Cariati, Francesca Bagnulo, Elena Scaglione, Consolata Carotenuto, Fabrizio Farina, Valeria D’Argenio, Francesca Carraturo, Paola D’Aprile, Mariateresa Vitiello, Ida Strina, Carlo Alviggi, Roberta Colicchio, Rossella Tomaiuolo, Paola Salvatore

**Affiliations:** 1 Department of Molecular Medicine and Medical Biotechnologies, University of Naples Federico II, via S. Pansini 5, 80131 Napoli, Italy; chiara.pagliuca@unina.it (C.P.); elena.scaglione@unina.it (E.S.); co.carotenuto@studenti.unina.it (C.C.); fr.carraturo@studenti.unina.it (F.C.); paodaprile@gmail.com (P.D.); mariateresa.vitiello2@unina.it (M.V.); roberta.colicchio@unina.it (R.C.); 2 CEINGE-Biotecnologie Avanzate, via G. Salvatore 486, 80145 Napoli, Italy; cariati@ceinge.unina.it (F.C.); dargenio@ceinge.unina.it (V.D.); 3 Fertility Unit, Maternal-Child Department, AOU Policlinico Federico II, 80131 Naples, Italy; f.bagnulo@studenti.unina.it (F.B.); ida.strina@unina.it (I.S.); carlo.alviggi@unina.it (C.A.); 4 CeSMA Centro Servizi Metrologici e Tecnologici Avanzati, University of Naples Federico II, Corso Nicolangelo Protopisani, 80146 Napoli, Italy; 5 Department of Law, Economics, Management and Quantitative Methods, University of Sannio, 82100 Benevento, Italy; f.farina@mediastat.it; 6 Department of Human Sciences and Quality of Life Promotion, San Raffaele Open University, via di Val Cannuta 247, 00166 Roma, Italy; 7 Department of Neuroscience, Reproductive Sciences and Odontostomatology, Federico II University, 80138 Naples, Italy; 8 Endocrinology and Experimental Oncology Institute (IEOS), National Research Council, 80131 Naples, Italy; 9 Università Vita-Salute San Raffaele, 20132 Milan, Italy; rossella.tomaiuolo@unina.it; 10 Task Force on Microbiome Studies, University of Naples Federico II, 80138 Naples, Italy

**Keywords:** urogenital infections, microbiological evaluation, sperm DNA fragmentation, male infertility

## Abstract

Fifteen percent of male infertility is associated with urogenital infections; several pathogens are able to alter the testicular and accessory glands’ microenvironment, resulting in the impairment of biofunctional sperm parameters. The purpose of this study was to assess the influence of urogenital infections on the quality of 53 human semen samples through standard analysis, microbiological evaluation, and molecular characterization of sperm DNA damage. The results showed a significant correlation between infected status and semen volume, sperm concentration, and motility. Moreover, a high risk of fragmented sperm DNA was demonstrated in the altered semen samples. Urogenital infections are often asymptomatic and thus an in-depth evaluation of the seminal sample can allow for both the diagnosis and therapy of infections while providing more indicators for male infertility management.

## 1. Introduction

**Figure 1:**
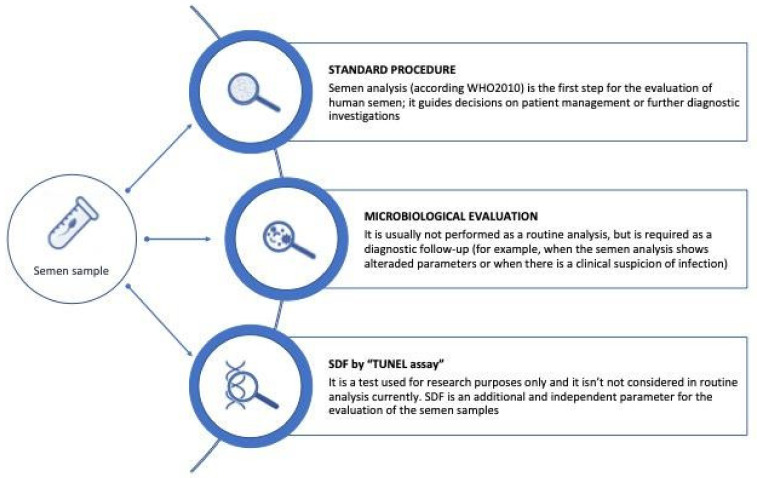
Methods used for the combined assessment of the human semen sample.

Nearly 190 million people struggle with infertility worldwide and male infertility accounts for 50% of couples’ infertility cases [1]. There are known pre-testicular (attributable to the dysfunction of the hypothalamic-pituitary axis), testicular (mainly testicular pathologies), and post-testicular (urogenital obstructions, vasectomy, and accessory glands impairment) causes that interfere with the composition of seminal fluid and the features of the spermatozoa [2]. In the other cases (30–50%) [1], it is assumed that this condition is determined by the coexistence of different or harder-to-identify factors (i.e., genetic disorders [3], environmental pollution [4], and infections [5]). In particular, male infertility is defined as unexplained when the parameters of the spermiogram are normal, and as idiopathic when the parameters of the spermiogram are altered without an identifiable cause [1]. The first step of the male fertility routine assessment is semen analysis; this evaluation does not discriminate between fertile or infertile men, but the alteration of some parameters indicates the need for further clinical investigation. Standard semen analysis provides, through a macroscopic (volume, pH, appearance, viscosity, and fluidification) and microscopic (concentration, motility, morphology and presence of non-sperm components) evaluation, data about sperm production and quality [6]. As the semen consists of a concentrated suspension of spermatozoa, stored in the epididymis and, at the time of ejaculation, diluted with the secretions of the accessory glands of the genital tract (mostly prostate and seminal vesicles), some parameters can reflect the sperm capacity of the testicle, the patency of the ejaculatory ducts (the total number of spermatozoa), and the secretory capacity of the accessory glands (the total fluid volume). Both macroscopic and microscopic parameters are considered to highlight inflammatory phenomena [6]. The presence of semen inflammation parameters can be determined by urogenital infections: La Vignera et al. reported an incidence of 13.8% of oligo-astheno-teratozoospermia (OAT) due to the presence of urogenital infections [7]. Infections of the male genitourinary tract account for about 15% of male infertility cases [8]. Infections, acute or chronic, may compromise spermatogenesis and sperm function. A positive semen culture can identify the type and severity of infection by quantifying the colony-forming units. The terms bacteriospermia and infection are distinguished from inflammation since the latter is the response of tissues to an infection [9]. Among infertile men, studies report a prevalence of infections between 11.6% and 45% in cases with a history of urethral discharge as a marker of infection [10,11]. Urogenital infections are also implicated in the pathogenetic mechanisms that alter the nemaspermic cell, such as increasing the percentage of spermatozoa with low mitochondrial membrane potential and apoptosis attributable to the cytotoxic effect exerted by bacteria through membrane permeabilization [12]. To date, a higher percentage of SDF (>30%) was found in infertile subjects compared with fertile subjects (approximately 5–15% SDF) [13,14,15,16,17]. In particular, SDF levels between 30% and 40% are negatively associated with sperm quality and SDF levels of >26% seem implicated in recurrent miscarriage [15,16,17]. Based on the observations that relate to the integrity of sperm DNA and the outcomes of pregnancy, the SDF evaluation in the diagnostic process of an infertile couple is becoming increasingly important. Therefore, it was proposed as an independent and additional parameter for assessing sperm quality and reproductive potential [14]. The purpose of our study was to evaluate if alterations in human semen parameters (1) are correlated to microbiological agents and (2) sperm DNA damage (Figure 1).

## 2. Material and Methods

### 2.1. Semen Samples Collection

In this study, 53 semen samples from men (aged 27–44 years) undergoing fertility investigations at the University of Naples Federico II during the period of 1 February 2019 to 31 January 2020 were included. Exclusion criteria for the study included primary gonadal pathologies and genital surgery, a history of radio and chemotherapy, primary or secondary hypogonadism, and concomitant therapies. The investigations were carried out following the rules of the Declaration of Helsinki (https://www.wma.net/what-we-do/medical-ethics/declaration-of-helsinki (accessed on 12 March 2021)). A written informed consent form was signed by all the participants involved in the study (Federico II Ethics Committee, Number: 382-18).

**Figure 2:**
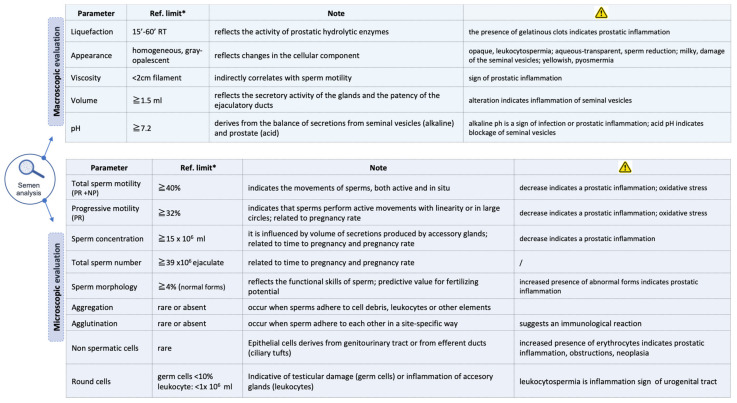
Semen analysis. Macroscopic and microscopic parameters used to evaluate the seminal sample. * The reference values refer to the lower 5th centiles (95% confidence interval) regarding the standards of conventional statistics applied to clinical chemistry.

The semen samples were collected between 2 and 7 days of sexual abstinence and the standard semen analysis was carried out according to the WHO protocol of 2010 [6] (Figure 2).

Semen samples were analyzed through macro- and microscopic evaluation using WHO’s methods at the time (see Supplementary Materials). Measurements were compared with the reference values, taken as reference to the cut-off at the lower 5th percentile, and all the alterations found were recorded.

### 2.2. Microbiological Evaluation

Before collecting seminal plasma, the patients proceeded with urine collection to better differentiate the infection of the seminal tract from urinary tract infection. About 1 mL of the semen sample was diluted (1:10) with sterile saline solution and centrifuged at 1500 rpm for 15 min at room temperature. After removing the supernatant, the sediment was resuspended in 100 µL of sterile saline solution or sterile saline solution with 10% glycerol. This procedure increases cultural sensitivity because it concentrates bacteria in the cell pellet and eliminates the seminal plasma, which can exert an inhibitory effect on bacterial growth. The cell pellet was spread on different culture media such as Becton Dickinson (BD) Trypticase Soy Agar with 5% sheep blood and McConkey agar for aerobic bacteria, BD Sabouraud Agar for fungi, BD Gardnerella Agar for searching *Gardnerella vaginalis*, and BD Chocolate agar for fastidious bacteria. All media were incubated at 37 °C. To evaluate viable bacteria, BD Trypticase Soy Agar, McConkey agar, and BD Sabouraud Agar were incubated under aerobic conditions for 24 h and 48 h, separately; to allow for the growth of *G. vaginalis* and fastidious bacteria, BD Gardnerella Agar and BD Chocolate Agar were incubated at 37 °C with 5% CO_2_ for 48 h. All clinical isolates were definitively identified by MALDI-TOF analysis [18].

Sexually transmitted pathogens, with fastidious growth requirements or non-cultivable characteristics, such as *Ureaplasma urealyticum*/*Ureaplasma. parvum*, *Mycoplasma hominis*/*Mycoplasma genitalium*, and *Trichomonas vaginalis*/*G. vaginalis*, were searched for in the seminal fluid by multiplex real-time PCR. The semen samples were equilibrated at room temperature, mixed by vortexing and 100 µL was pre-treated with a lysis reagent buffer. The DNA was extracted from the specimens using a RealLine DNA-Express kit (Bioron Diagnostics GmbH, Romerberg, Germany) following the manufacturer’s instructions, and stored frozen at −20 °C until testing. The detection of mycoplasmas, ureaplasmas, and *T. vaginalis*/*G. vaginalis* was performed by employing RealLine STI Pathogen Kits (BIORON Diagnostics), multiplex real-time PCR assays for the qualitative detection of sexually transmitted infection (STI) pathogen DNA. The RT-PCR tests were performed according to the manufacturer’s protocol. The amplification was performed in a CFX96 Real-Time thermocycler (Bio-Rad, Hercules, CA, USA). Each PCR was performed with 50 µL of extracted DNA. The thermal cycle conditions consisted of an initial incubation at 50 °C for 2 min, pre-denaturation at 95 °C for 2 min, followed by 50 cycles of alternating incubations: denaturation at 94 °C for 10 s, and annealing and extension at 60 °C for 40 s. Samples were considered positive with an average cycle threshold (Ct) value of ≤40, except for *U. parvum* and *G. vaginalis*, which had a Ct value of ≤32. For the detection of *Chlamydia trachomatis/Neisseria gonorrhoeae* DNA, the Xpert^®^ CT/NG system (Cepheid, Sunnydale, CA, USA), fully automated real-time PCR test, was used.

The evaluation of the infectious status was standardized, following the instruction of Calogero et al. [19]. In details, based on the etiological agent, each was assigned a score: for sexually transmitted agents (STAs), a score of 3 was assigned; Gram-negative bacteria, a score of 2; Gram-positive bacteria, a score of 1; and for the presence of commensal flora, no score was assigned. The bacterial load was scored as follows: severe bacterial load (>10^4^ CFU/mL), a score of 3; moderate bacterial load (10^3^–10^4^ CFU/mL), a score of 2; mild bacterial load (>/=10^3^ CFU/mL), a score of 1; and reduced bacterial load (<10^3^ CFU/mL), a score of 0. The sum of the scores (etiological agent plus bacterial load) determined the infective value for the analyzed semen samples; if the obtained value was greater than 3, the sample was defined infected; otherwise, if the value was equal to or lower than 3, the semen was defined as not infected.

### 2.3. Sperm DNA Fragmentation Analysis

The sperm DNA fragmentation (SDF) analysis was carried out for each sample by a TUNEL assay using an Apo-Direct kit (BD Pharmingen, San Diego, CA, USA) through a flow cytometer. The step-by-step approach to the measurement of sperm DNA fragmentation was carried out as follows: an aliquot containing 5 × 10^6^/ mL sperm (control and patients) was pipetted into each tube. Once removed, the seminal plasma spermatozoa were fixed in 1.0 mL of paraformaldehyde (1%) in ice for 30 min. Samples were washed in phosphate-buffered saline (PBS) (centrifuge at 300× *g* for 7′) and the pellets were resuspended with 1 mL of ice-cold ethanol (70%) in ice for at least 30′. A total of 2 mL of both negative and positive assay controls, provided in the kit, was aliquoted in duplicate. All samples were centrifuged at 300× *g* for 7′. The supernatant was carefully removed by aspiration without disturbing the cell pellet. Following this, 1.0 mL of wash buffer was added to each tube, vortexed, and centrifuged twice. The staining solution was prepared according to the manufacturer’s instructions. Once prepared, 50 μL of the staining solution was added to all the tubes before being covered with aluminum foil and incubated for 60′ at 37 °C. At the end of the incubation period, 1.0 mL of rinse buffer was added to each tube, and the mixture was centrifuged at 300× *g* for 7′. The supernatant was then discarded and this step was repeated. The samples were analyzed by a flow cytometry equipped with a 488 nm argon laser as the light source. Two dyes were used: propidium iodide (PI) for total DNA staining and fluorescein isothiocyanate-2′-deoxyuridine-5-triphosphate (FITC-dUTP) for fragmented DNA staining. Each sample was run in duplicate and cells positive for TUNEL were defined as those containing fragmented DNA. The results are expressed as the percentage of sperm with DNA fragmentation (%SDF) using the flow cytometer software. A minimum of 10,000 events were recorded.

### 2.4. Statistical Analysis

The parametric T-test on the comparison between means was used to evaluate the statistically significant differences between the semen samples of the Test and Control groups with respect to the study parameters. Pearson’s correlation coefficient was used to measure the linear correlation between the sperm parameters and infective status, or %SDF, of the Test and Control groups. A chi^2^ test was applied to calculate the %SDF significance between the groups analyzed and the odds ratio was calculated with 95% confidence.

## 3. Results

The samples were divided into two populations based on the presence or absence of an alteration of the parameters as revealed by the standard semen analysis.

Among the 53 enrolled subjects, 37 were reported to have altered parameters at semen analysis (Test group) and 16 had no semen alteration (Control group).

**Figure 3:**
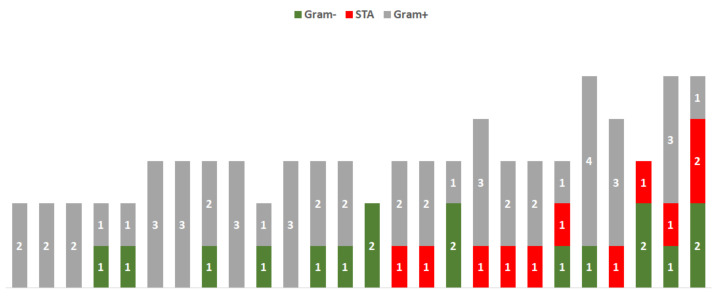
Prevalence of infectious agents in semen samples belonging to the Test group. Each histogram represents a single semen sample: the presence of Gram-positive bacteria was found in 24 samples (in gray), 13 Gram-negative (in green), and 10 sexually transmitted agents (STAs, in red).

The microbiological evaluation showed that 70.2% (26/37) of the analyzed semen samples belonging to the Test group presented with multiple microbial agents (values greater than three), as reported in Figure 3. The analysis revealed Gram-negative bacteria in 13 samples, sexually transmitted agents in 10 samples, and the presence or co-presence of Gram-positive bacteria in 24 samples (Figure 3).

**Figure 4:**
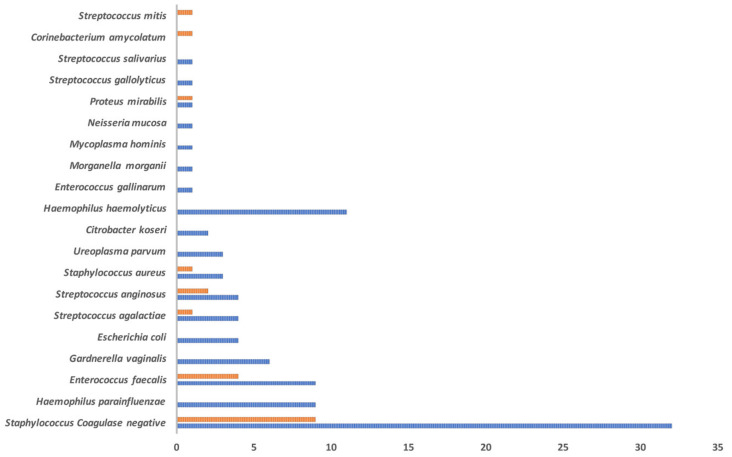
Microbiological evaluation of the collected semen samples among the Test (blue histogram; 37 samples) and the Control (orange histogram; 16 samples) groups. Histograms are used to report the number of samples positive for each microorganism.

In the Control group, just 31.2% (5/16) of the samples presented with an infective value greater than three. The microbiological agents identified in the two analyzed groups are reported in Figure 4.

Next, in order to highlight if the infective status is related to specific semen alterations, a correlation analysis was carried out. In the Test group, the statistical analysis showed a significant negative correlation between infectious status, semen volume, and total sperm concentration (*p*-value 0.038 and 0.014, respectively) (Table 1). Moreover, a significant correlation was found between the infective status and seminal motility. In detail, the infective status was significantly inversely correlated with specific motility parameters, such as PR + NP% and PR% (*p*-value 0.005 and 0.008, respectively) (Table 1).

**Figure 5:**
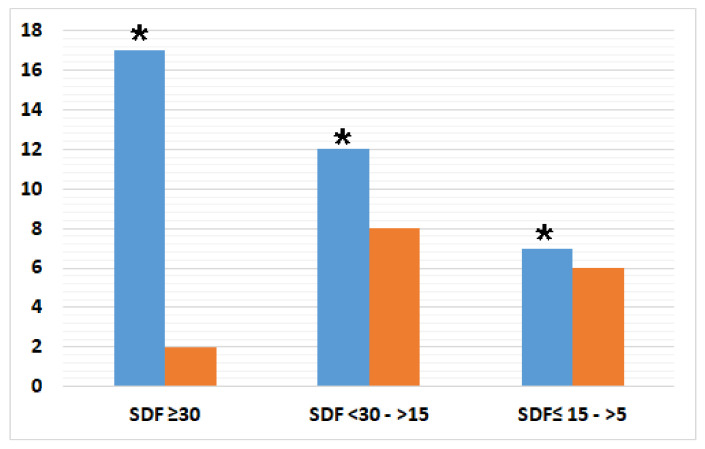
The sperm DNA fragmentation analysis on semen samples within the Test (blue histogram) and Control (orange histogram) groups. The significant difference was calculated through C\the chi^2^ test (*p*-value 0.049). *p*-value < 0.05, *.

The same analysis was carried out for the Control group and no correlation was found between the semen parameters and infection status. The sperm DNA fragmentation analysis for the Test group showed that 47.2% (17/36) had an SDF greater than or equal to 30%, 33.3% (12/36) had a %SDF between 15% and 30%, and 19.4% (7/36) had an SDF between 5% and 15%. The same analysis carried out on semen samples from the Control group showed that 12.5% (2/16) had an SDF greater than or equal to 30%, 50% (8/16) had an SDF between 15% and30%, and 37.5% (6/16) had an SDF between 5% and 15% (Figure 5). Semen samples belonging to the Test group were six times more at risk of a high degree of DNA fragmentation than Control groups with a %SDF level higher than 30% (odds ratio (OR) 5.95, 95% CI, 1.18–29.96) Table 2. Moreover, the semen samples with microbial agents showed a higher percentage of SDF compared with negative samples (Supplementary Figure S1).

The correlation between the sperm parameters and SDF is shown in Table 3: in samples of the Test group, a significant negative correlation of pH parameter (*p*-value 0.028), motility PR % (*p*-value 0.036) and motility NP % (*p*-value 0.047) was found. No significant correlation was found between the SDF and the semen parameters of samples belonging to the Control group (Table 3).

## 4. Discussion

The etiopathogenetic mechanisms that determine potential damage to spermatozoa are numerous and act at different levels: sperm cells can be damaged directly (with evidence of sperm alterations ranging from concentration, motility, morphology to DNA fragmentation, mitochondrial function, apoptosis, and acrosomal reaction) by pathogenic germs [20], bacterial products [21], toxic metabolites produced by microorganisms [21], seminal leukocytes, and soluble factors, such as reactive oxygen species (ROS) and cytokines [22]. In particular, oxidative stress during sperm transport through the male reproductive tract is likely the most frequent cause of sperm DNA damage [23,24].

However, it is not possible to deduce if there is damage of the spermatozoa from the mere presence of leukocytes or other parameters warning of inflammatory status [25,26,27,28,29]. Therefore, in order to achieve the aim of the study, 53 semen samples from subjects undergoing fertility investigation were subjected to standard, microbiological, and sperm DNA fragmentation analysis.

Infection status with a score greater than three was revealed in 70% of semen samples with altered parameters. The results showed that infections deeply affect sperm motility, concentration, and volume. Therefore, dysbiosis in the male reproductive tract microbiota can lead to seminal abnormalities.

Accordingly, in previous studies, bacterial virulence factors detected in human semen samples were reported to deteriorate semen quality by triggering a local inflammatory reaction [30,31].

The correlation between infected status and motility can be explained by the action of cytokines, which are able to modulate and regulate immune and inflammatory responses and to modify the behavior of other cells, inducing new activities such as growth, differentiation, and apoptosis [32]. The molecules most involved in infertility are interleukin (IL) -1, IL-2, IL-6, IL-8, interferon-ɣ, and tumor necrosis factor-α (TNF-α) [33]. TNF-α was shown to reduce sperm motility and increase the percentage of spermatozoa with in vitro early and late apoptosis indices.

The inflammatory response of the genitourinary tract to the invasion of microorganisms is known to activate the release of leukocytes and inflammatory mediators (ROS and cytokines) that affect sperm DNA integrity and negatively influence fertility [34]. Accordingly, in the group of semen with altered parameters and positive infections, a risk six times higher to have a high degree of DNA fragmentation was found. Moreover, considering the presence of microbial agents in semen samples, the %SDF was higher than semen samples without microorganisms.

Leukocytospermia can lead to an overproduction of ROS, which, through the phenomenon of lipid peroxidation of the membranes, alter lipids, proteins, and DNA, thus damaging the sperm membrane and mitochondria, with consequent alterations in motility and sperm DNA [27]. Further sources of ROS in semen are the sperm, particularly immature sperm with cytoplasmic retention and abnormal head morphology characterized by the retention of residual cytoplasm [28]. Both leukocytospermia and the retention of residual cytoplasm within the sperm were associated with increased sperm DNA damage, likely secondary to an increased level of ROS produced by these cells [29].

Since urogenital infections are often asymptomatic, the microbiological investigation is useful for completing the assessment of male infertility in the presence of altered semen sample parameters [35]. Furthermore, in our study, 31.2% of the analyzed samples within the Control population (i.e., with no alteration of semen parameters) had an infective score greater than 3. It is likely the detected infection was of recent acquisition, so no altered seminal parameters were found in association, nor was there an increased percentage of spermatozoa with fragmented DNA equal to or greater than 15% [36].

Sperm DNA integrity was considered an additional predicting factor of male fertility since infertile subjects showed a high percentage of fragmented DNA [15,16,17,18].

In particular, results showed that 47.2% of semen samples from the Test group had an SDF greater than 30% vs. the 12.5% of the Control group. According to literature data, high levels of sperm DNA damage were correlated with poor seminal parameters such as motility [37,38,39]. However, reports revealed that the standard semen analysis produces normal results in 15% of male factor infertility cases [40].

In addition, discordant results were observed when examining the relationship between fragmented DNA testing and in vitro fertilization (IVF) success rates [41]. A systematic review and meta-analysis in 2016 analyzing 30 studies showed that fragmented DNA testing had limited ability to predict pregnancy in assisted reproductive techniques, especially between IVF and intracytoplasmic sperm injection (ICSI). Other similar articles showed decreased rates of IVF success with higher fragmented DNA [42,43,44]. An SDF >30%, especially >40%, is often considered a direct cause of reproductive failure, including in IVF and ICSI outcomes. Moreover, a >40% SDF is considered a risk factor of spontaneous abortion [45]. Moreover, since the IVF techniques do not occur in a sterile environment, bacteria can affect semen and embryo quality, resulting in a worse clinical outcome [46].

Considering what was mentioned above, DNA integrity can be an indicator for preimplantation genetic testing [47,48].

The predictive value of sperm DNA fragmentation tests depends on several factors, some related to the damage to the sperm (for example, percentage of damaged sperm, extent of DNA damage per sperm, and combination of DNA fragmentation and nucleotide damage) and others related to the ability of the oocyte to repair damage to the sperm DNA (the oocyte can repair single-stranded damage, while double-stranded damage is irreversible) [49,50]. Despite the known relationship between SDF and semen quality parameters, the lack of a standardized method for evaluating DNA damage in a routine diagnostic setting limits its use in the assessment of fertility. An integrated approach based on standard analysis and microbiological evaluation of semen samples, as proposed in the present study, can suggest an alteration in DNA integrity in patients with suspected infertility.

## 5. Conclusions

These data underline the importance of a thorough evaluation of couples with unexplained infertility and the need to focus on male factors. In particular, it is necessary to consider semen microbiological status, as these infections are often featured by a paucisymptomatic course. As a consequence, infections affecting the genitourinary tract are often diagnosed too late, after they have already spread to one or more accessory sex glands, thereby becoming chronic and more difficult to eradicate.

The importance of following up on the alterations found in semen analysis, infection status, and DNA fragmentation is evident and can help drive eventual treatments and characterize undiagnosed and unexplained infertility.

## Figures and Tables

**Table 1:** Correlation between the sperm parameters of the Test group and infective status.

Parameters	Correlation Coefficient	*p*-Value
pH	0.046	0.790
Volume	**−0.346**	0.038 *
Conc (×10^6^ mL)	−0.223	0.192
Conc/tot	**−0.404**	0.014 *
Motility PR + NP (%)	**−0.460**	0.005 **
Motility PR (%)	**−0.437**	0.008 **
Leucocytes (1 × 10^6^/mL)	0.185	0.280
Normal forms (%)	−0.258	0.135

PR: rapid progressive; NP: non-progressive. The statistical significance of the correlation coefficient was evaluated by a T-test student (*p*-value < 0.05, *; *p*-value < 0.01, **).

**Table 2:** Risk of sperm DNA fragmentation on Test semen samples vs. Control semen samples.

%SDF Levels	Person’s Chi Squared	*p*-Value	OR (95%CI)
≥30	0.020	0.029	5.95 (1.18–29.96)
<30–≥15	1.926	0.165	0.40 (0.11–1.49)
≤15–>5	1.300	0.254	0.50 (0.15–1.66)

OR: Odd’s ratio calculated with a 95% confidential interval.

**Table 3:** Correlation between sperm parameters and %SDF in semen samples from the Test and Control groups.

	Test Group		Control Group	
Parameters	SDF Tunel	*p*-Value	SDF Tunel	*p*-Value
pH	**−0.362**	0.028 *	−0.067	0.806
Volume	0.228	0.174	−0.005	0.987
Conc (×10^6 mL^)	0.031	0.857	−0.277	0.298
Conc/tot	0.114	0.501	0.083	0.761
Motility PR + NP (%)	**−0.336**	0.042 *	0.272	0.309
Motility PR (%)	**−0.346**	0.036 *	0.322	0.224
Leucocytes (1 × 10^6^/^mL^)	−0.037	0.827	−0.214	0.426
Normal forms (%)	−0.190	0.268	−0.177	0.512

PR: rapid progressive; NP: non-progressive. Student’s *t*-test was used to evaluate the statistical significance of the correlation coefficient (*p*-value < 0.05, *).

## Data Availability

The data presented in this study are available on request from the corresponding author.

## Supplementary Materials

The following are available online at https://www.mdpi.com/article/10.3390/genes12050654/s1, Figure S1: Difference of %SDF between semen samples with and without microbial agents.

## Abbreviations

OAT	oligo-astheno-teratozoospermia
Ct	cycle threshold
FITC	-dUTP fluorescein isothiocyanate-2′-deoxyuridine-5-triphosphate
IL	interleukin
ICSI	intracytoplasmic sperm injection
IVF	in vitro fertilization
NP	non-progressive
PBS	phosphate-buffered saline
PI	propidium iodide
PR	rapid progressive
ROS	reactive oxygen species
%SDF	percentage of sperm DNA fragmentation
SDF	sperm DNA fragmentation
STA	sexually transmitted agents
STI	sexually transmitted infection
TNF-α	tumor necrosis factor-α

## References

[1] Agarwal A., Parekh N., Panner Selvam M.K., Henkel R., Shah R., Homa S.T., Ramasamy R., Ko E., Tremellen K., Esteves S. (2019). Male Oxidative Stress Infertility (MOSI): Proposed Terminology and Clinical Practice Guidelines for Management of Idiopathic Male Infertility. World J. Men’s Health.

[2] Krausz C. (2011). Male infertility: Pathogenesis and clinical diagnosis. Best Pract. Res. Clin. Endocrinol. Metab..

[3] Cariati F., D’Argenio V., Tomaiuolo R. (2019). The evolving role of genetic tests in reproductive medicine. J. Transl. Med..

[4] Cariati F., D’Uonno N., Borrillo F., Iervolino S., Galdiero G., Tomaiuolo R. (2019). Bisphenol a: An emerging threat to male fertility. Reprod. Biol. Endocrinol..

[5] Tomaiuolo R., Veneruso I., Cariati F., D’Argenio V. (2020). Microbiota and Human reproduction: The case of male infertility. High Throughput.

[6] World Health Organization (2010). WHO Laboratory Manual for the Examination and Processing of Human Semen.

[7] Vicari E., Calogero A.E., Condorelli R.A., Vicari L.O., La Vignera S. (2012). Male accessory gland infection frequency in infertile patients with chronic microbial prostatitis and irritable bowel syndrome: Transrectal ultrasound examination helps to understand the links. J. Androl..

[8] Pellati D., Mylonakis I., Bertoloni G., Fiore C., Andrisani A., Ambrosini G., Armanini D. (2008). Genital tract infections and infertility. Eur. J. Obstet. Gynecol. Reprod. Biol..

[9] Solomon M., Henkel R. (2017). Semen culture and the assessment of genitourinary tract infections. Indian J. Urol..

[10] Nieschlag E., Behre H. (1997). Andrology: Male Reproductive Health and Dysfunction.

[11] Bayasgalan G., Naranbat D., Tsedmaa B., Tsogmaa B., Sukhee D., Amarjargal O., Lhagvasuren T., Radnaabazar J., Rowe P.J. (2004). Clinical patterns and major causes of infertility in Mongolia. J. Obstet. Gynaecol. Res..

[12] Sellami H., Znazen A., Sellami A., Mnif H., Louati N., Zarrouk S.B., Keskes L., Rebai T., Gdoura R., Hammami A. (2014). Molecular detection of Chlamydia trachomatis and other sexually transmitted bacteria in semen of male partners of infertile couples in Tunisia: The effect on semen parameters and spermatozoa apoptosis markers. PLoS ONE.

[13] Moazenchi M., Totonchi M., Salman Yazdi R., Hratian K., Mohseni Meybodi M.A., Ahmadi Panah M., Chehrazi M., Mohseni Meybodi A. (2018). The impact of Chlamydia trachomatis infection on sperm parameters and male fertility: A comprehensive study. Int. J. STD AIDS.

[14] Sergerie M., Laforest G., Bujan L., Bissonnette F., Bleau G. (2005). Sperm DNA fragmentation: Threshold value in male fertility. Hum. Reprod..

[15] Osman A., Alsomait H., Seshadri S., El-Toukhy T., Khalaf Y. (2015). The effect of sperm DNA fragmentation on live birth rate after IVF or ICSI: A systematic review and meta-analysis. Reprod. Biomed. Online.

[16] Simon L., Zini A., Dyachenko A. (2017). A systematic review and meta-analysis to determine the effect of sperm DNA damage on in vitro fertilization and intracytoplasmic sperm injection outcome. Asian J. Androl..

[17] Evenson D.P., Wixon R. (2008). Data analysis of two in vivo fertility studies using Sperm Chromatin Structure Assay-derived DNA fragmentation index vs. pregnancy outcome. Fertil. Steril..

[18] Cicatiello A.G., Iula D.V., Pagliuca C., Pastore G., Pagliarulo C., Catania M.R., Colicchio R., Picardi M., Raia V., Salvatore P. (2014). Identification of *Inquilinus limosus* in cystic fibrosis: A first report in Italy. N. Microbiol..

[19] La Vignera S., Condorelli R.A., Vicari E., Salmeri M., Morgia G., Favilla V., Cimino S., Calogero A.E. (2014). Microbiological investigation in male infertility: A practical overview. J. Med. Microbiol..

[20] Ochsendorf F.R. (2008). Sexually transmitted infections: Impact on male fertility. Andrologia.

[21] Grande G., Milardi D., Baroni S., Luca G., Pontecorvi A. (2018). Identification of seminal markers of male accessory gland inflammation: From molecules to proteome. Am. J. Reprod. Immunol..

[22] Barroso G., Morshedi M., Oehninger S. (2000). Analysis of DNA fragmentation, plasma membrane translocation of phosphatidyl- serine and oxidative stress in human spermatozoa. Hum. Reprod..

[23] Aitken R.J., De Iuliis G.N. (2010). On the possible origins of DNA damage in human spermatozoa. Mol. Hum. Reprod..

[24] Sakkas D., Alvarez J.G. (2010). Sperm DNA fragmentation: Mechanisms of origin, impact on reproductive outcome, and analysis. Fertil. Steril..

[25] La Vignera S. (2012). Male accessory gland infections: Anatomical extension of inflammation and severity of symptoms evaluated by an original questionnaire. Andrologia.

[26] La Vignera S., Vicari E., Condorelli R., D’Agata R., Calogero A.E. (2011). Hypertrophic-congestive and fibro-sclerotic ultrasound variants of male accessory gland infection have different sperm output. J. Endocrinol. Investig..

[27] Lanzafame F.M., La Vignera S., Vicari E., Calogero A.E. (2009). Oxidative stress and medical antioxidant treatment in male infertility. Reprod. Biomed. Online.

[28] Ollero M., Gil-Guzman E., Lopez M.C., Sharma R.K., Agarwal A., Larson K., Evenson D., Thomas A.J., Alvarez J.G. (2001). Characterization of subsets of human spermatozoa at different stages of maturation: Implications in the diagnosis and treatment of male infertility. Hum. Reprod..

[29] Erenpreiss J., Hlevicka S., Zalkalns J., Erenpreisa J. (2002). Effect of leukocytospermia on sperm DNA integrity: A negative effect in abnormal semen samples. J. Androl..

[30] Fraczek M., Kurpisz M. (2015). Mechanisms of the harmful effects of bacterial semen infection on ejaculated human spermatozoa: Potential inflammatory markers in semen. Folia Histochem. Cytobiol..

[31] Fujita Y., Mihara T., Okazaki T., Shitanaka M., Kushino R., Ikeda C., Negishi H., Liu Z., Richards J.S., Shimada M. (2011). Toll-like receptors (TLR) 2 and 4 on human sperm recognize bacterial endotoxins and mediate apoptosis. Hum. Reprod..

[32] Schuppe H.C., Meinhardt A., Allam J.P., Bergmann M., Weidner W., Haidl G. (2008). Chronic orchitis: A neglected cause of male infertility?. Andrologia.

[33] Perdichizzi A., Nicoletti F., La Vignera S., Barone N., D’Agata R., Vicari E., Calogero A.E. (2007). Effects of tumour necrosis factor-alpha on human sperm motility and apoptosis. J. Clin. Immunol..

[34] Farsimadan M., Motamedifar M. (2020). Bacterial infection of the male reproductive system causing infertility. J. Reprod. Immunol..

[35] Korrovits P., Ausmees K., Mändar R., Punab M. (2008). Prevalence of asymptomatic inflammatory (National Institutes of Health Category IV) prostatitis in young men according to semen analysis. Urology.

[36] La Vignera S., Condorelli R.A., Vicari E., Tumino D., Morgia G., Favilla V., Cimino S., Calogero A.E. (2013). Markers of semen inflammation: Supplementary semen analysis?. J. Reprod. Immunol..

[37] Irvine D.S., Twigg J.P., Gordon E.L., Fulton N., Milne P.A., Aitken R.J. (2000). DNA integrity in human spermatozoa: Relationships with semen quality. J. Androl..

[38] Lopes S., Sun J.G., Jurisicova A., Meriano J., Casper R.F. (1998). Sperm deoxyribonucleic acid fragmentation is increased in poor-quality semen samples and correlates with failed fertilization in intra- cytoplasmic sperm injection. Fertil. Steril..

[39] Muratori M., Piomboni P., Baldi E., Filimberti E., Pecchioli P., Moretti E., Gambera L., Baccetti B., Biagiotti R., Forti G. (2000). Functional and ultrastructural features of DNA- fragmented human sperm. J. Androl..

[40] Agarwal A., Allamaneni S.S. (2005). Sperm DNA damage assessment: A test whose time has come. Fertil. Steril..

[41] Miller D., Vukina J. (2020). Recent advances in clinical diagnosis and treatment of male factor infertility. Postgrad. Med..

[42] Cissen M., Wely M.V., Scholten I., Mansell S., Bruin J.P., Mol B.W., Braat D., Repping S., Hamer G. (2016). Measuring Sperm DNA Fragmentation and Clinical Outcomes of Medically Assisted Reproduction: A Systematic Review and Meta-Analysis. PLoS ONE.

[43] Simon L., Castillo J., Oliva R., Lewis S.E. (2011). Relationships between human sperm protamines, DNA damage and assisted reproduction outcomes. Reprod. Biomed. Online.

[44] Li Z., Wang L., Cai J., Huang H. (2006). Correlation of sperm DNA damage with IVF and ICSI outcomes: A systematic review and meta-analysis. J. Assist. Reprod. Genet..

[45] Evenson D.P. (2017). Evaluation of sperm chromatin structure and DNA strand breaks is an important part of clinical male fertility assessment. Transl. Androl. Urol..

[46] Štšepetova J., Baranova J., Simm J., Parm Ü., Rööp T., Sokmann S., Korrovits P., Jaagura M., Rosenstein K., Salumets A. (2020). The complex microbiome from native semen to embryo culture environment in human in vitro fertilization procedure. Reprod. Biol. Endocrinol..

[47] Cariati F., Savarese M., D’Argenio V., Salvatore F., Tomaiuolo R. (2017). The SEeMORE strategy: Single-tube electrophoresis analysis-based genotyping to detect monogenic diseases rapidly and effectively from conception until birth. Clin. Chem. Lab. Med..

[48] D’Argenio V., Nunziato M., D’Uonno N. (2017). Indicazioni e limiti della diagnosi genetica preimpianto. Bioch. Clinic..

[49] Rusz A., Pilatz A., Wagenlehner F., Linn T., Diemer T., Schuppe H.C., Lohmeyer J., Hossain H., Weidner W. (2011). Influence of urogenital infections and inflammation on semen quality and male fertility. World J. Urol..

[50] Simon L., Emery B., Carrell D.T. (2019). Sperm DNA Fragmentation: Consequences for Reproduction. Adv. Exp. Med. Biol..

